# The Use of Sucroferric Oxyhydroxide Prior to Sigmoidoscopy in Patients With End-Stage Kidney Disease: A Case Report

**DOI:** 10.1177/20543581241273998

**Published:** 2024-09-12

**Authors:** Jennifer Horwitz, Katelyn Roberts, Stephanie Canning, Douglas Mcintosh, Deborah Zimmerman

**Affiliations:** 1 Faculty of Medicine, University of Ottawa, ON, Canada; 2 Department of Medicine, The Ottawa Hospital, ON, Canada; 3 Department of Gastroenterology, The Ottawa Hospital, ON, Canada; 4 The Ottawa Hospital, ON, Canada; 5 Department of Nephrology, The Ottawa Hospital, ON, Canada

**Keywords:** sucroferric oxyhydroxide, bowel preparation, hyperphosphatemia, kidney transplantation, phosphate binder

## Abstract

**Rationale:**

Sucroferric oxyhydroxide is an iron-based phosphate-binding medication that has been approved for the treatment of hyperphosphatemia in patients with end-stage kidney disease. Given the low overall iron release from the polynuclear iron(III)-oxyhydroxide molecule, recommendations regarding its use prior to colonoscopy/sigmoidoscopy have not been developed.

**Presenting concerns of the patient:**

A 51-year-old male with a known history of end-stage renal disease treated with hemodialysis was referred to Gastroenterology for consideration of colonoscopy to rule out malignancy because of a history of rectal bleeding. This was to be completed prior to proceeding with a living-donor kidney transplant.

**Diagnoses:**

Flexible sigmoidoscopy done after non-diagnostic colonoscopy demonstrated diffuse “charcoal-like” material that prevented adequate visualization of the bowel despite standard bowel preparation. The findings were believed to be secondary to the use of sucroferric oxyhydroxide prescribed for hyperphosphatemia.

**Interventions:**

The patient was subsequently instructed to discontinue sucroferric oxyhydroxide for 2 weeks prior to his repeat sigmoidoscopy procedure.

**Outcomes:**

The patient’s repeat sigmoidoscopy after discontinuing sucroferric oxyhydroxide allowed for adequate bowel visualization that revealed only a benign lipoma.

**Teaching Points:**

This case demonstrates the potential for sucroferric oxyhydroxide use to result in poor bowel preparation and resulting inadequate visualization on lower gastrointestinal endoscopy. It serves to highlight the clinical implications leading to the need for repeated procedures, which contributes to resource waste and unnecessary costs to the healthcare system, as well as delays in diagnostic evaluation required for transplantation; patient frustration was evident.

## Abrege

**Justification:**

L’oxyhydroxyde sucro-ferrique, un médicament à base de fer liant le phosphate, a été approuvé pour le traitement de l’hyperphosphatémie chez les patients atteints d’insuffisance rénale terminale. La molécule polynucléaire fer (lll) — oxyhydroxyde ne libérant qu’une faible quantité globale de fer, aucune recommandation n’a été développée concernant son utilisation avant une coloscopie/sigmoïdoscopie.

**Présentation du cas:**

Un homme de 51 ans connu pour insuffisance rénale terminale et traité par hémodialyse a été orienté en gastroentérologie pour subir une coloscopie afin d’exclure une tumeur maligne en raison d’antécédents de saignement rectal. L’examen devait être complété avant de procéder à la greffe de rein par donneur vivant.

**Diagnostic:**

Une sigmoïdoscopie souple réalisée après une coloscopie non diagnostique a révélé une matière diffuse de type « charbon de bois » qui empêchait de bien voir l’intestin malgré une préparation intestinale adéquate. Ce résultat a été jugé secondaire à l’utilisation d’oxyhydroxyde sucro-ferrique prescrit pour traiter l’hyperphosphatémie.

**Intervention:**

On a demandé au patient d’interrompre le traitement par oxyhydroxyde sucro-ferrique pendant deux semaines avant de répéter la procédure de sigmoïdoscopie.

**Résultats:**

La sigmoïdoscopie répétée après l’arrêt de l’oxyhydroxyde sucroferrique a permis une visualisation adéquate de l’intestin qui n’a révélé qu’un lipome bénin.

**Enseignements tirés:**

Ce cas démontre que l’utilisation d’oxyhydroxyde sucro-ferrique peut entraîner une mauvaise préparation intestinale et entraver la visualisation lors d’une endoscopie gastro-intestinale basse. Il met en évidence les répercussions cliniques qui justifient des procédures répétées, lesquelles contribuent au gaspillage des ressources et entraînent des coûts inutiles pour le système de santé, ainsi que les retards dans l’évaluation diagnostique requise pour la transplantation; dans ce cas, la frustration du patient était évidente.

## Introduction

Hyperphosphatemia is one of the hallmark features of end-stage kidney disease (ESKD) and has been associated with morbidity and mortality in this patient population. Elevated serum phosphate levels are associated with cardiovascular disease, vascular calcification,^1^ and hyperparathyroidism. Secondary hyperparathyroidism is associated with bone and muscular pain and an increased risk of fracture.^2^ As such, the Kidney Disease: Improving Global Outcomes (KDIGO) guidelines recommend lowering serum phosphate levels toward the normal range with a low phosphate diet, dialysis, and oral phosphate binders when appropriate.^3^

Several classes of phosphate binders exist and are available for use in clinical practice including calcium and non–calcium-based phosphate binders. Sucroferric oxyhydroxide is an example of a newly available iron-containing phosphate binder that is prescribed to patients with ESKD for the management of hyperphosphatemia. Unlike other oral iron supplements, in vitro studies have demonstrated that the active moiety of sucroferric oxyhydroxide, polynuclear iron(III)-oxyhydroxide, is practically insoluble and poorly absorbed by the body, leading to minimal overall iron absorption.^4^ Rather, the drug binds to phosphate within the aqueous environment of the gastrointestinal tract, and the bound phosphate is eliminated within the feces. Given the low iron release by the polynuclear iron(III)-oxyhydroxide, no specific recommendations regarding sucroferric oxyhydroxide use prior to endoscopic procedures exist. Specifically, the drug manufacturers of sucroferric oxyhydroxide have not suggested that the drug should be stopped 5 to 7 days prior to colonoscopy as is standard of care with oral iron supplements.

The clinical significance of this logistical question is highlighted by the not-uncommon situation in which a patient taking sucroferric oxyhydroxide requires a lower gastrointestinal endoscopy. This is of particular significance in the setting of a pre-transplant workup for patients being considered for kidney transplantation. The Canadian Kidney Transplant Guidelines recommend that patients being evaluated for kidney transplantation be screened for pre-transplant malignancy, including colorectal cancer, according to the most up-to-date clinical practice guidelines developed for the general population.^5^ As such, any patient over the age of 50 years with a positive fecal immunochemical test (FIT) or with a history of unexplained rectal bleeding requires a colonoscopy or sigmoidoscopy to rule out malignancy prior to kidney transplant. Recommendations regarding the use of sucroferric oxyhydroxide prior to colonoscopy are therefore essential for appropriate patient management.

Herein, we describe a case of sucroferric oxyhydroxide use leading to inadequate endoscopic evaluation of the bowel in a patient undergoing sigmoidoscopy as part of a pre-transplant workup.

## Presenting Concerns

A 51-year-old male with ESKD treated with hemodialysis had a known history of unexplained rectal bleeding for several months, for which he had been referred to Gastroenterology for investigation. He had 2 non-diagnostic colonoscopies and an abnormal computed tomography (CT) colonography. Further investigation was required to rule out malignancy and as part of his living-donor kidney transplant workup. The specific timeline of events for the patient’s clinical course is outlined in Table 1.

**Table 1.** Timeline of Events for the Patient.

Date	Relevant information and pertinent results
January 2022	Patient was referred to the gastrointestinal service for pre-transplant workup given his history of rectal bleeding.
September 2022	The patient undergoes initial colonoscopy with adequate visualization of bowel; 2 small polyps are removed. Unable to fully advance to cecum.
January 2023	Started new medication (sucroferric oxyhydroxide) for the management of hyperphosphatemia.
February 2023	The patient undergoes follow-up flexible sigmoidoscopy. Charcoal-like material is seen throughout the large bowel during the procedure.
April 2023	The patient undergoes repeat flexible sigmoidoscopy after stopping sucroferric oxyhydroxide. Satisfactory views were obtained.
October 2023	The patient underwent successful living-donor kidney transplant.

## Clinical Findings

The patient first endorsed intermittent rectal bleeding in January 2022. Despite having advanced chronic kidney disease, his ferritin (270 µg/L), iron saturation (37%), and hemoglobin (126 g/L) were reasonable at that time without the use of iron supplements or erythropoietin stimulating agents. The patient was referred to gastroenterology for consideration of a lower gastrointestinal endoscopy to rule out malignancy. He had his first colonoscopy in February 2022, but bowel preparation was inadequate; a polyp was removed at 25 cm.

**Figure 1.**
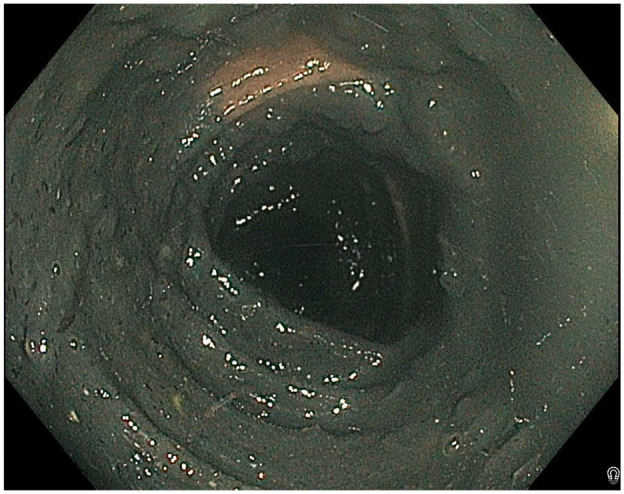
Retained black residues seen on flexible sigmoidoscopy.

The patient started hemodialysis in July 2022 and had a repeat colonoscopy in September 2022 after being treated with twice daily polyethylene glycol sachets and pre-procedure liquid polyethylene glycol (RestoraLAX, Colyte); 2 polyps were removed, and a presumed lipoma was identified in the distal sigmoid, but the scope could not be advanced to the cecum in spite of multiple attempts at repositioning. The patient underwent CT colonography to better visualize the cecum; a 7-mm polyp was identified in the mid-sigmoid colon. To evaluate this finding, the patient was scheduled for a follow-up flexible sigmoidoscopy. Unfortunately, this procedure provided limited evaluation of the bowel due to inadequate bowel preparation. Namely, the gastroenterologist described the presence of “retained solid charcoal-like material” (Figure 1) seen throughout the colon. This was despite having had 6 tap water enemas prior to the procedure (chosen as bowel preparation by the gastroenterologist, given the patient’s history of hyperphosphatemia) as well as repeated attempts at suctioning intra-procedurally. The patient was advised that he would require a repeat sigmoidoscopy in order to better evaluate the colonic mucosa.

## Diagnostic Focus and Assessment

Importantly, it was noted that in the interim time period between the patient’s most recent colonoscopy and his follow-up flexible sigmoidoscopy, he had been prescribed sucroferric oxyhydroxide 500 mg 3 times daily with meals starting January 2023. This was for the management of worsening hyperphosphatemia and hyperparathyroidism despite high doses of calcium carbonate. The patient was not provided with specific instructions to hold this medication and therefore continued to take it in the days leading up to the procedure. The patient did, however, endorse having dark black stools since starting this medication. During this time, he was not receiving intravenous or oral iron for anemia management. Given this clinical history and the known iron-based formulation of sucroferric oxyhydroxide, it was determined to be the cause of the charcoal-like material seen during sigmoidoscopy.

## Therapeutic Focus and Assessment

**Figure 2.**
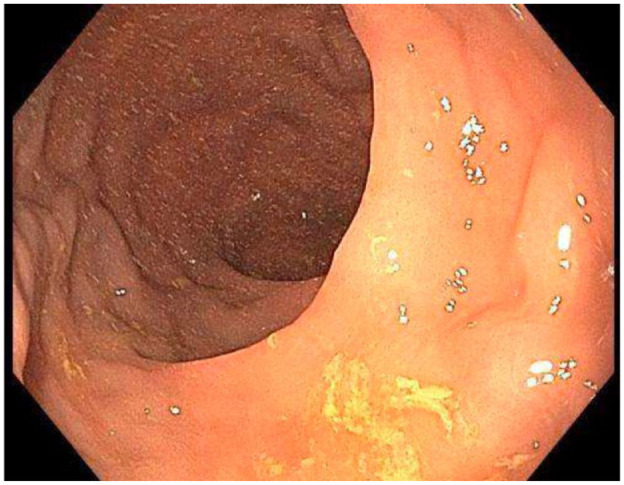
Repeat sigmoidoscopy with adequate views.

The patient was subsequently scheduled for a repeat sigmoidoscopy and instructed to replace his sucroferric oxyhydroxide with a calcium-based phosphate binder for 2 weeks prior to the procedure. On the follow-up sigmoidoscopy, the charcoal-like substance was no longer seen, and the colon was adequately visualized (Figure 2). The colonic mass in the mid-sigmoid was again visualized and determined to be a lipoma; the remainder of the sigmoidoscopy was reported as normal apart from diverticulosis.

## Follow-up and Outcomes

The patient subsequently underwent a successful kidney transplant from a living-related donor in October 2023. Despite this good outcome, he expressed understandable frustration about further delays in the living-donor transplant surgery created by the need for repeat sigmoidoscopy due to an inadequate bowel preparation secondary to a medication prescribed by his kidney team. He was likewise frustrated about how these outcomes could have been avoided if he had been provided with the proper initial instructions to discontinue his sucroferric oxyhydroxide use prior to his first sigmoidoscopy.

## Discussion

To our knowledge, this is the first published case report of sucroferric oxyhydroxide use causing dark residues and obscured views during colonoscopy or sigmoidoscopy. To support this claim, we conducted a literature search with the assistance of a health sciences librarian to search MEDLINE and EMBASE (OVID) for articles related to sucroferric oxyhydroxide use and endoscopy. This search was run on January 30, 2024. The search strategy included terms reflecting gastrointestinal endoscopy and sucroferric oxyhydroxide. Endoscopy search terms included variations of *colonoscopy, sigmoidoscopy, endoscopy*, and *colon capsule endoscopy.* Sucroferric oxyhydroxide search terms included *sucroferric oxyhydroxide, ferric oxide*, and *iron sucrose.* The search yielded 15 total articles between the 2 databases; however, on further review, none were relevant to our case. A copy of the complete search strategy is available in the Supplemental Material.

An Internet search reveals only one other mention of this informally on Twitter/X, in which a New York-based gastrointestinal pathologist described the peculiar presence of dark pigmentation seen throughout the colon in a patient with kidney failure treated with hemodialysis and sucroferric oxyhydroxide.^6^ Interestingly, this physician also included pathology slide images that clearly depict iron staining within the intestinal cells.

Sucroferric oxyhydroxide’s degradation product, mononuclear iron species, can be released from the drug’s surface and absorbed, although minimally. The iron uptake from radiolabeled sucroferric oxyhydroxide was investigated by the manufacturer in both chronic kidney disease (CKD) and healthy patients with low iron stores. The median uptake of radiolabeled iron in the blood 21 days after the administration of a 2000-mg dose was 0.43% in healthy subjects and 0.04% in patients with CKD. However, the lack of significant iron absorption from sucroferric oxyhydroxide does not translate into a lack of iron-related complications such as dark-colored feces that has the potential to negatively impact the adequacy of bowel preparation for procedures such as colonoscopy. In our case, the use of sucroferric oxyhydroxide led to the requirement for a repeated procedure that was inconvenient for the patient, had the potential to delay a living-donor kidney transplant, and incurred unnecessary costs to the hospital.

## Patient Perspective

This drug was offered to me by the Physician Assistant in the dialysis clinic at the Ottawa Hospital, Riverside Campus. I was informed that this was a newer drug that worked very well. Unfortunately, it was not covered by my insurance company, but the Physician Assistant worked her magic, and the drug was offered free of charge as it was very expensive. From my understanding it was paid for by the drug company. I had almost zero side effects other than the black stool. When I went for the colonoscopy, I was informed the colon was stained black and the doctor was unable to see, thus, I was told to discontinue the drug until after the colonoscopy and was told by dialysis that the colon could stay stained up to 6 months after discontinued use, which was very frustrating as the colonoscopy was holding me back from the transplant. Luckily after a few weeks my stool came back to regular colour, and I was scheduled for another colonoscopy. The doctor was able to see into the colon as it was no longer stained and was told everything was normal and she would see me in 3 years.

## Supplemental Material

sj-docx-1-cjk-10.1177_20543581241273998 – Supplemental material for The Use of Sucroferric Oxyhydroxide Prior to Sigmoidoscopy in Patients With End-Stage Kidney Disease: A Case Report Supplemental material, sj-docx-1-cjk-10.1177_20543581241273998 for The Use of Sucroferric Oxyhydroxide Prior to Sigmoidoscopy in Patients With End-Stage Kidney Disease: A Case Report by Jennifer Horwitz, Katelyn Roberts, Stephanie Canning, Douglas Mcintosh and Deborah Zimmerman in Canadian Journal of Kidney Health and Disease
