# Supplementary material for: The Use of Sucroferric Oxyhydroxide Prior to Sigmoidoscopy in Patients With End-Stage Kidney Disease: A Case Report
Source: Can J Kidney Health Dis. 2024 Sep 12;11:20543581241273998. doi: 10.1177/20543581241273998 (PMC11406624; doi:10.1177/20543581241273998)
Supplement: sj-docx-1-cjk-10.1177_20543581241273998 – Supplemental material for The Use of Sucroferric Oxyhydroxide Prior to Sigmoidoscopy in Patients With End-Stage Kidney Disease: A Case Report [file sj-docx-1-cjk-10.1177_20543581241273998.docx]

**Supplemental Material:**

**Medline (OVID) Search Strategy:**

#1 colonoscopy [tw]

#2 endoscopy [tw]

#3 sigmoidoscopy [tw]

#4 Endoscopy, Gastrointestinal [MESH] OR Colonoscopy [MESH] OR Sigmoidoscopy

#5 OR 1 – 4

#6 Ferric Oxide, Saccharacted [MESH]

#7 “sucroferric oxyhydroxide” [tw] OR “sucroferic oxyhydroxide” [tw] OR “iron sucrose” [tw] or “velphoro” [tw]

#8 “iron sucrose” [tw] OR “Iron sugar” [tw] OR “Saccharacted iron” [tw] OR “Saccharacted iron oxide” [tw] OR “Sucroferric oxyhydroxide” [tw] OR “Saccharacted ferric oxide” [tw] OR “Ferrivenin” [tw] OR “Venoferrum” [tw] OR “Encifer” [tw] OR “Feojectin” [tw] OR “Ferijet” [tw] OR “Hippiron” [tw] OR “Proferrin” [tw] OR “Sucrofer” [tw] OR “Velphoro” [tw] OR “Iviron” [tw] OR “Fesin” [tw] OR “Ferum Hausmann” [tw] OR “Ferrum Vitis” [tw] OR “Neo-ferrum” [tw] OR “Ferosoft S” [tw] OR “Fe-back” [tw] OR “Ferplex SS” [tw] OR “Fe-lib” [tw] OR “Colliron I.V.” [tw] OR “Ferrum Hausmann i.v.” [tw] OR “Iron oxide, saccharated” [tw] OR “Succharated ferric oxide” [tw] OR “Ferric oxide, saccharacted” [tw] OR “UNII-FZ7NYF5N8L" [tw] OR "FZ7NYF5N8L" [tw] OR "IRON OXIDE SACCHARATED" [tw] OR "EINECS 232-464-7" [tw] OR "NSC 27278" [tw] OR "XI921" [tw] OR "XI-921" [tw] OR "DB09146" [tw] OR “Ferric saccharate adj5 iron oxide” [tw] OR "Q27888379" [tw]

#9 6 OR 7 OR 8

#10 5 AND 9

**Embase (OVID) Search Strategy:**

#1 colonoscopy [tw]

#2 endoscopy [tw]

#3 sigmoidoscopy [tw]

#4 intestine endoscopy [Emtree] OR sigmoidoscopy [Emtree]

#5 colonoscopy [Emtree] OR colon capsule endoscopy [Emtree] OR ileocolonoscopy [Emtree]

#6 OR 1 – 4

#7 “sucroferric oxyhydroxide” [tw] OR “sucroferic oxyhydroxide” [tw] OR “iron sucrose” [tw] OR velphoro [tw]

#8 “Iron sucrose” [tw] OR "Iron sugar" [tw] OR "Saccharated iron" [tw] OR "Saccharated iron oxide" [tw] OR "Sucroferric oxyhydroxide" [tw] OR "Saccharated ferric oxide" [tw] OR "Ferrivenin" [tw] OR "Venoferrum" [tw] OR "Encifer" [tw] OR "Feojectin" [tw] OR "Ferijet" [tw] OR "Hippiron" [tw] OR "Proferrin" [tw] OR "Sucrofer" [tw] OR "Velphoro" [tw] OR "Iviron" [tw] OR "Fesin" [tw] OR "Ferum Hausmann" [tw] OR "Ferrum Vitis" [tw] OR "Neo-ferrum" [tw] OR "Ferosoft S" [tw] OR "Fe-back" [tw] OR "Ferplex SS" [tw] OR "Fe-lib" [tw] OR "Colliron I.V." [tw] OR "Ferrum Hausmann i.v." [tw] OR "Iron oxide, saccharated" [tw] OR "Succharated ferric oxide" [tw] OR "Ferric oxide, saccharated" [tw] OR "UNII-FZ7NYF5N8L" [tw] OR "FZ7NYF5N8L" [tw] OR "IRON OXIDE SACCHARATED" [tw] OR "EINECS 232-464-7" [tw] OR "NSC 27278" or "XI921" [tw] OR "XI-921" [tw] OR "DB09146" [tw] OR “Ferric saccharate adj5 iron oxide)” [tw] OR “Q27888379" [tw]

#9 sucroferric oxyhydroxide [Emtree]

#10 pa21 [tw] OR “pa 21” [tw]

#11 iron saccharate [Emtree]

#12 OR 7 – 12

#13 6 AND 12
